# Supplementary material for: Nuclear-embedded mitochondrial DNA sequences in 66,083 human genomes
Source: Nature. 2022 Oct 5;611(7934):105–14. doi: 10.1038/s41586-022-05288-7 (PMC9630118; doi:10.1038/s41586-022-05288-7)
Supplement: Supplementary file 1 — This file contains supplementary results, references for the supplementary results and Supplementary Figs. 1–9. [file 41586_2022_5288_MOESM1_ESM.docx]

**Supplementary Results**

**NUMT associated with phenotypes**

*Rare Disease*

There were 75 unsolved individuals who had a rare or ultra-rare (F <1% in whole cohort) NUMT in the coding sequence of a PanelApp gene (29 different genes)^1^. Comparison of the participant phenotypes and disorders associated with the genes did not reveal many good candidates for the NUMTs being causative of the disease. 13 individuals had a NUMT in six genes with no established gene-disease association (*HIVEP1, PVR, CLASP1, HTR3C, ZNF714, GNA14*) and these were not investigated further.

*Genes causing recessive disorders*

We considered the phenotype match and whether there was parental consanguinity because there is an increased likelihood of homozygous variants in consanguineous families. The bioinformatic pipeline was not able to distinguish between heterozygous and homozygous NUMTs because it was primarily based on detecting discordant reads.

31 patients had a NUMT in an autosomal recessive disease gene and their phenotype did not match the reported features of the disorder. These were discounted as being causative (in genes *CRADD*, *SMG8, STEAP3, DSG3, TF, TTLL5, ITPKB, RHOH, SPARC, ADAMTS9, CYBA* and *KIAA0825*).

Two participants with a rare NUMT in the *CRADD* gene had phenotypes which could potentially match (one had intellectual disability and one had seizures and intellectual disability) but were not specific to the related genetic disorder. Biallelic pathogenic variants in *CRADD* cause intellectual disability with megalencephaly and a mild variant of lissencephaly^2^. Neither had a second rare variant in the *CRADD* gene, and their parents were not consanguineous so these were considered unlikely to be diagnostic.

A proband with inherited white matter disorders had a NUMT in *PNPLA6*. Biallelic pathogenic variants in *PNPLA6* are associated with a spectrum of neurogenic disorders including Boucher-Neuhausen syndrome, Oliver-McFarlane syndrome, Laurence-Moon syndrome and spastic paraplegia 39^3^ but there was no second rare variant in this individual. Furthermore, there was no consanguinity and there was an autosomal dominant family history.

A proband with intellectual disability and his unaffected father had a NUMT in the 3’-UTR of the *SLC3A1* gene. Monoallelic or biallelic mutations in *SLC3A1* cause cystinuria^4^, which did not match the phenotype in this family.

*Genes causing disorders by mechanisms other than haploinsufficiency*

Five individuals had a NUMT in a gene which is reported to cause disease by a mechanism other than loss of function, such as missense variants only (*RPS20, WNK4, WDFY3, TNC*).

*NUMT insertion is in an intronic or upstream region*

Four individuals had a NUMT in the *KCNQ2* gene. Loss of function and missense mutations in *KCNQ2* cause epilepsy^5^, but the phenotypes of the individuals were very varied (congenital hearing impairment, craniosynostosis, pulmonary fibrosis and primary ciliary dyskinesia). Review of the insertion coordinates showed it was in an intron of *KCNQ2*, but did overlap an exon 1 of AL353658.1 (a gene with no known disease association).

A NUMT near the *TBCE* gene (which causes an autosomal recessive, severe developmental disorder^6^) was observed in nine individuals from five families, all of South-Asian ethnicity. However, the insertion site was upstream of the gene.

One proband has a NUMT inserted in the *SMARCA4* gene intron region. The *SMARCA4* gene is highly intolerant of loss of function variation (pLI=1)^7^. Missense mutations with gain of function or dominant-negative effects are reported as a relatively uncommon cause of Cofin Siris syndrome^8^. Loss of function variants are reported in rhabdoid tumour predisposition syndrome^9^ which may also present with small cell carcinoma of the ovary, hypercalcaemic type^10^. The proband in this study was a 50-year-old man with cardiomyopathy, with no documented history of malignancy or features of Cofin Siris syndrome (such as intellectual disability, dysmorphic features or hypoplastic 5^th^ finger nails).

One proband has a NUMT inserted into the *AFF3* gene intron region. The *AFF3* gene is highly intolerant of loss of function variation (pLI =1)^7^. Patients with de novo missense mutations and one patient with a de novo intragenic deletion have been reported with KINSSHIP syndrome^11^. The clinical features include horseshoe kidney, mesomelic dysplasia, seizures, hypertrichosis, intellectual disability and pulmonary involvement. The proband in this study was a 38-year-old woman with seizures but no congenital anomalies or developmental delay.

*NUMT insertion in an exon*

Four members of a consanguineous family had a ~700bp NUMT insertion in the final (7^th^) exon of the *PPP2CA* gene. The family was recruited because two siblings had developmental glaucoma. The parents were unaffected. *De novo* loss of function and missense variants are reported to cause an autosomal dominant neurodevelopmental disorder with mild to profound intellectual disability, language delay, hypotonia, epilepsy and brain abnormalities^12^. We hypothesise that the NUMT insertion in the final exon is not disrupting the gene function because the region is predicted to escape nonsense mediated decay.

There were no genomic medicine centre diagnoses which were explained by NUMT insertions.

There was no significant difference in the number of NUMTs in people with mtDNA maintenance disorders^13^, compared to the rest of the cohort.

**Molecular evolution of NUMT sequences**

150 NUMT variants were present in the majority of individuals carrying exactly the same NUMT, so likely arose before the NUMT insertion. In keeping with this, the ratio of non-synonymous (Ka) to synonymous (Ks) variants (Ka/Ks) of these 150 variants was no different to the Ka/Ks for real mtDNA estimated from the same 41,050 individuals (150 NUMT variants Ks/Ka = 0.42, real mtDNA Ka/Ks = 0.49, *P* = 0.55, 95% CI = 0.72-2.04, Fisher Exact Test) (**Fig. 6a** - left). This analysis endorsed our classification and allowed the definition 5637 variants which likely arose after NUMT insertion into the nuclear genome (NUMT-specific variants) (**Methods**). We studied NUMT-specific variants in three categories using increasingly stringent filtering criteria to determine whether the filtering had a major impact on our interpretation. The categories were as follows: Total group A = all 5637 NUMT-specific variants; Subgroup B = 3838 NUMT-specific variants seen in more than one individual on the same NUMT; and high-stringency Subgroup C = 99 NUMT-specific variants present in more than one individual and also validated in another member of the same family when possible. Six of the 5637 NUMT-specific variants corresponded to known pathogenic mtDNA mutations in humans^14^: 8993G, 12706C, 13042A, 13051A, 13094C and 14849C, listed according to the original mtDNA site. Long-read sequencing showed that all were within single copy NUMTs, making them unlikely to cause high levels of pseudo-heteroplasmy leading to a false diagnosis of mtDNA disease^15^. The Ka/Ks of the NUMT-specific variants was greater than real mtDNA (Total group A = 3.2, *vs* mtDNA *P* = 0, odds ratio=6.6, 95% CI = 6.0-7.2; Subgroup B = 3.5, *vs.* mtDNA *P* = 0, odds ratio=7.1, 95% CI = 6.4-8.0; Subgroup C: 2.1 *vs.* real mtDNA *P* = 1.73 x 10^-6^, odds ratio=4.2, 95% CI = 2.2-8.2; Fisher Exact Test) (**Fig. 6a**). Moreover, Ka/Ks of Subgroup C was no different to the null hypothesis for random mutations of 2.91 (*P* = 0.25, odds ratio=1.20, 95% CI = 0.72-2.64, Fisher Exact Test) (**Fig. 6a**). Therefore, once translocated to the nucleus, the same genomic sequence is no longer under evolutionary constraint, consistent with NUMTs having no functional role.

The trinucleotide mutational signatures of NUMT-specific variants from all three groups were different to real mtDNA^16^ (**Fig. 6b**) (total group A vs real mtDNA, *P* = 0; subgroup B vs real mtDNA, *P* = 0; subgroup C vs real mtDNA, *P* = 9.1 x 10^-15^, Fisher's combined p value method). The signatures of total group A and subgroup B were no different to the expected random mutations (total group A vs expected, *P* = 0.1; subgroup B vs expected, *P* = 0.05, Kolmogorov-Smirnov test). When compared to cancer mutational signatures^17^, the signatures of NUMT-specific variants showed different correlation patterns to real mtDNA (**Fig. 6c**). Although the mutational signature for subgroup C more closely resembled real mtDNA, there were differences, particularly within the C>T transition group (**Fig. 6b**). However, despite extensive NUMT CpG methylation (**Fig. 3b, c**, **Extended Data Fig.3e**), we did not see enrichment for C>T transitions affecting CpG sites which are the hallmark of 5-methylcytosine modifications as recently proposed for Arabidopsis^18^. Thus, different mutational mechanisms operate on the same sequence of DNA after its insertion into the nuclear genome.

**References for the Supplementary Results**

1. Martin, A.R. *et al.* PanelApp crowdsources expert knowledge to establish consensus diagnostic gene panels. *Nat Genet* **51**, 1560-1565 (2019).

2. Di Donato, N. *et al.* Mutations in CRADD Result in Reduced Caspase-2-Mediated Neuronal Apoptosis and Cause Megalencephaly with a Rare Lissencephaly Variant. *Am J Hum Genet* **99**, 1117-1129 (2016).

3. Synofzik, M. *et al.* PNPLA6 mutations cause Boucher-Neuhauser and Gordon Holmes syndromes as part of a broad neurodegenerative spectrum. *Brain* **137**, 69-77 (2014).

4. Calonge, M.J. *et al.* Cystinuria caused by mutations in rBAT, a gene involved in the transport of cystine. *Nat Genet* **6**, 420-5 (1994).

5. Singh, N.A. *et al.* A novel potassium channel gene, KCNQ2, is mutated in an inherited epilepsy of newborns. *Nat Genet* **18**, 25-9 (1998).

6. Parvari, R. *et al.* Mutation of TBCE causes hypoparathyroidism-retardation-dysmorphism and autosomal recessive Kenny-Caffey syndrome. *Nat Genet* **32**, 448-52 (2002).

7. Karczewski, K.J. *et al.* The mutational constraint spectrum quantified from variation in 141,456 humans. *Nature* **581**, 434-443 (2020).

8. Sekiguchi, F. *et al.* Genetic abnormalities in a large cohort of Coffin-Siris syndrome patients. *J Hum Genet* **64**, 1173-1186 (2019).

9. Schneppenheim, R. *et al.* Germline nonsense mutation and somatic inactivation of SMARCA4/BRG1 in a family with rhabdoid tumor predisposition syndrome. *Am J Hum Genet* **86**, 279-84 (2010).

10. Ramos, P. *et al.* Small cell carcinoma of the ovary, hypercalcemic type, displays frequent inactivating germline and somatic mutations in SMARCA4. *Nat Genet* **46**, 427-9 (2014).

11. Voisin, N. *et al.* Variants in the degron of AFF3 are associated with intellectual disability, mesomelic dysplasia, horseshoe kidney, and epileptic encephalopathy. *Am J Hum Genet* **108**, 857-873 (2021).

12. Reynhout, S. *et al.* De Novo Mutations Affecting the Catalytic Calpha Subunit of PP2A, PPP2CA, Cause Syndromic Intellectual Disability Resembling Other PP2A-Related Neurodevelopmental Disorders. *Am J Hum Genet* **104**, 357 (2019).

13. Viscomi, C. & Zeviani, M. MtDNA-maintenance defects: syndromes and genes. *J Inherit Metab Dis* **40**, 587-599 (2017).

14. Ratnaike, T.E. *et al.* MitoPhen database: a human phenotype ontology-based approach to identify mitochondrial DNA diseases. *Nucleic Acids Res* **49**, 9686-9695 (2021).

15. Wei, W. *et al.* Nuclear-mitochondrial DNA segments resemble paternally inherited mitochondrial DNA in humans. *Nat Commun* **11**, 1740 (2020).

16. Wei, W., Gomez-Duran, A., Hudson, G. & Chinnery, P.F. Background sequence characteristics influence the occurrence and severity of disease-causing mtDNA mutations. *PLoS Genet* **13**, e1007126 (2017).

17. Sondka, Z. *et al.* The COSMIC Cancer Gene Census: describing genetic dysfunction across all human cancers. *Nat Rev Cancer* **18**, 696-705 (2018).

18. Fields, P.D. *et al.* Complete sequence of a 641-kb insertion of mitochondrial DNA in the Arabidopsis thaliana nuclear genome. *Genome Biol Evol* **14**, evac059 (2022).

**Supplementary Figures**

**
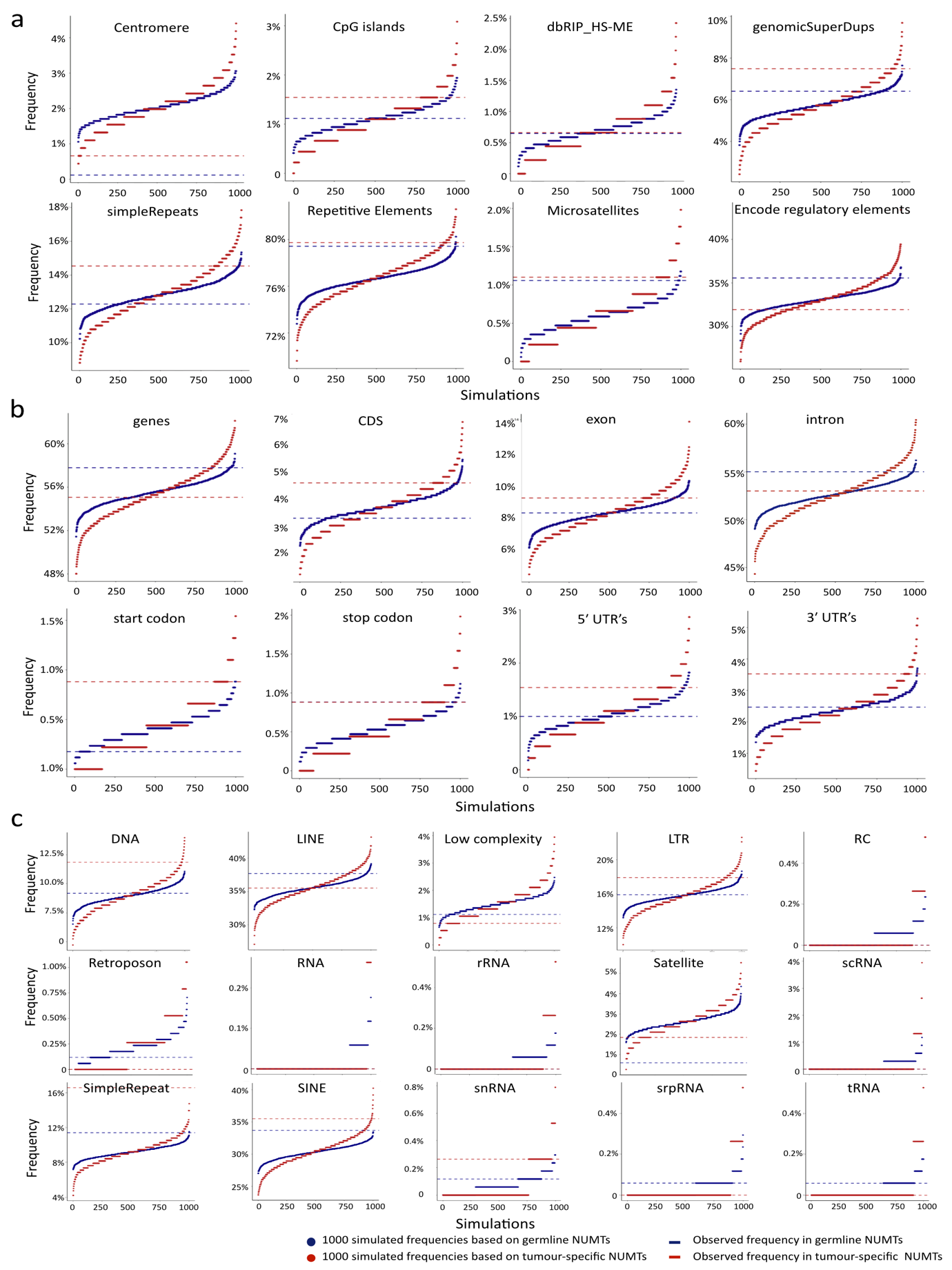
**

**Supplementary Figure 1.** **Enrichment analysis of NUMT insertions in the nuclear genome regions**. Germline NUMTs and tumour-specific NUMTs shown in the different colours.

**
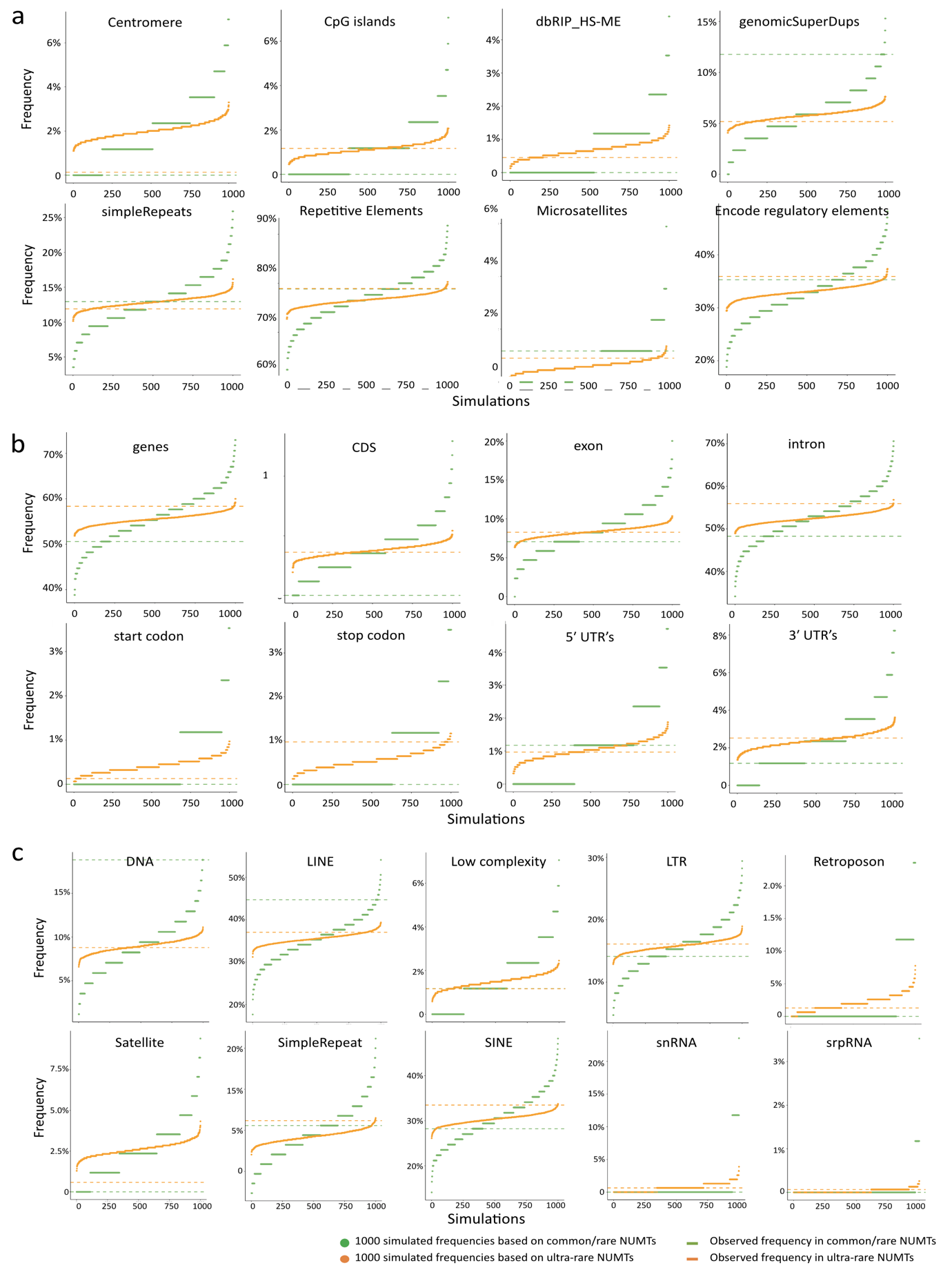
**

**Supplementary Figure 2. Enrichment analysis of NUMT insertions in the nuclear genome regions.** Germline common & rare NUMTs, and germline ultra-rare NUMTs shown in the different colours.

**
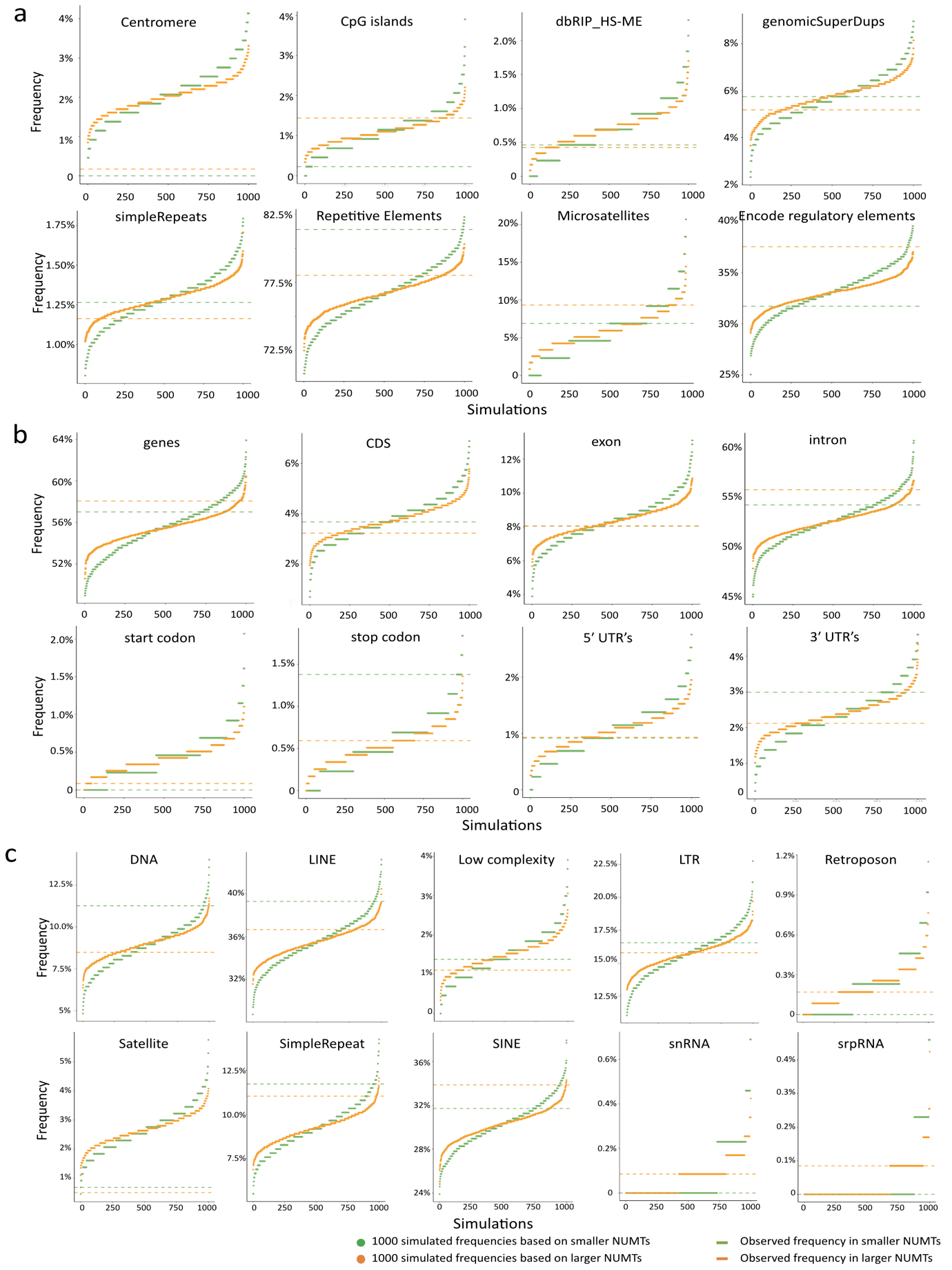
**

**Supplementary Figure 3. Enrichment analysis of NUMT insertions in the nuclear genome regions.** Germline larger NUMTs and germline smaller NUMTs shown in the different colours.


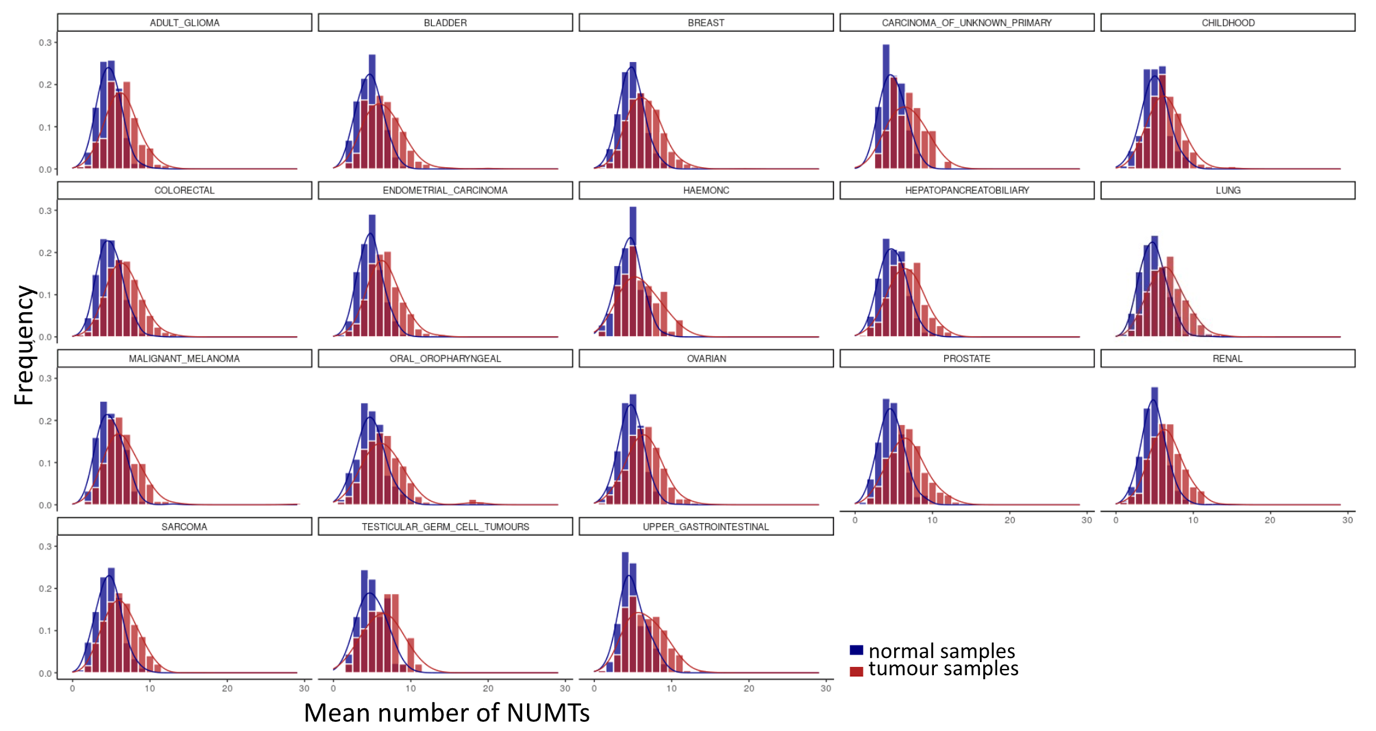


**Supplementary Figure 4. Histogram of the average number of total NUMTs detected within each cancer type.** The cancer types with sample size > 45 shown only.


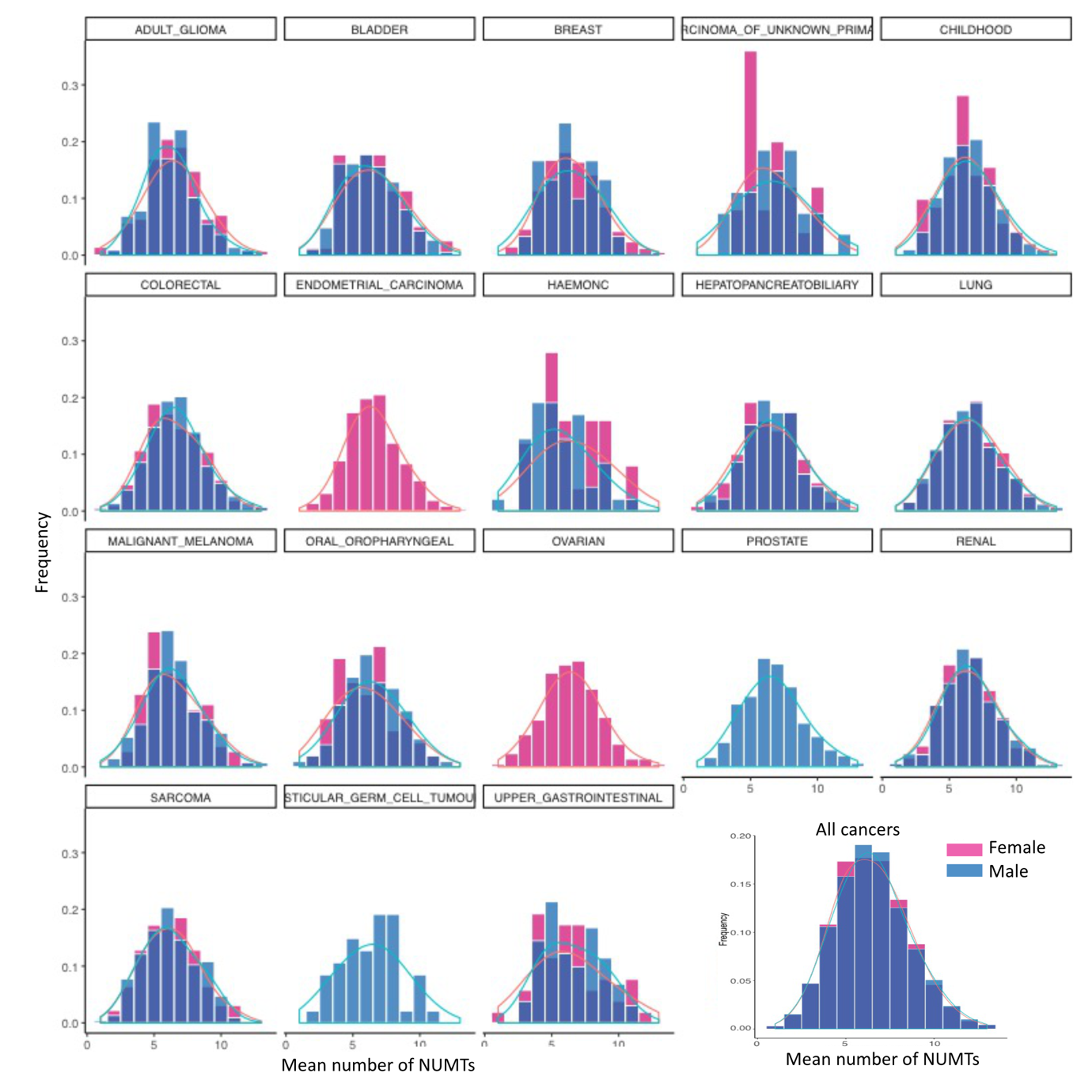


**Supplementary Figure 5. Histogram of the average number of total NUMTs detected within each cancer type and all cancer types (bottom right).** Male and female shown in different colours.

**
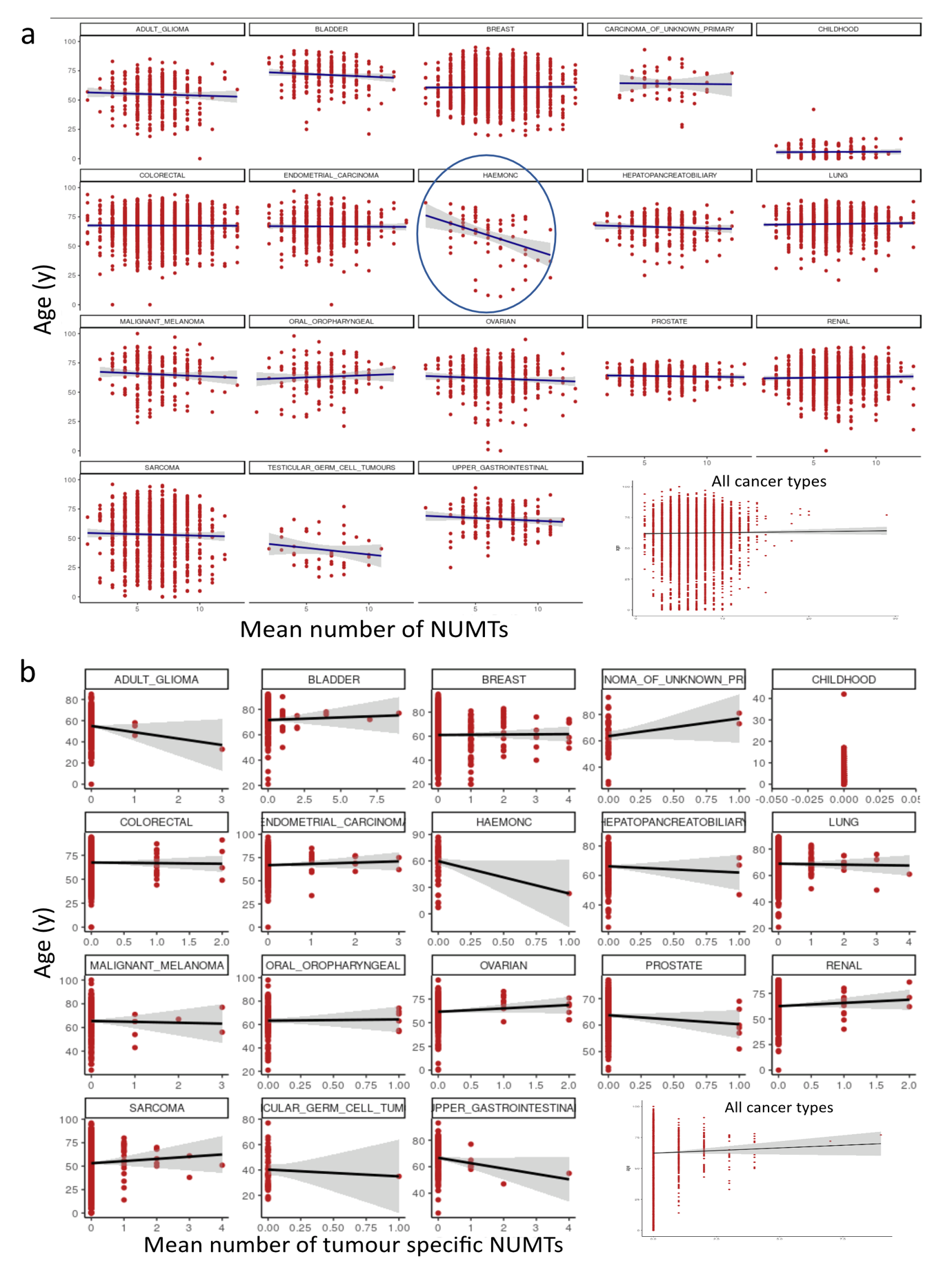
**

**Supplementary Figure 6. Correlation of the average number of NUMTs and donor’s age. a.** Correlation of the average number of total NUMTs and donor’s age within each cancer type and all cancer types (bottom right). **b.** Correlation of the average number of tumour-specific NUMTs and donor’s age within each cancer type and all cancer types (bottom right). Only the cancer types with sample size > 45 are shown. The shaded band represents 95% confidence interval.

**
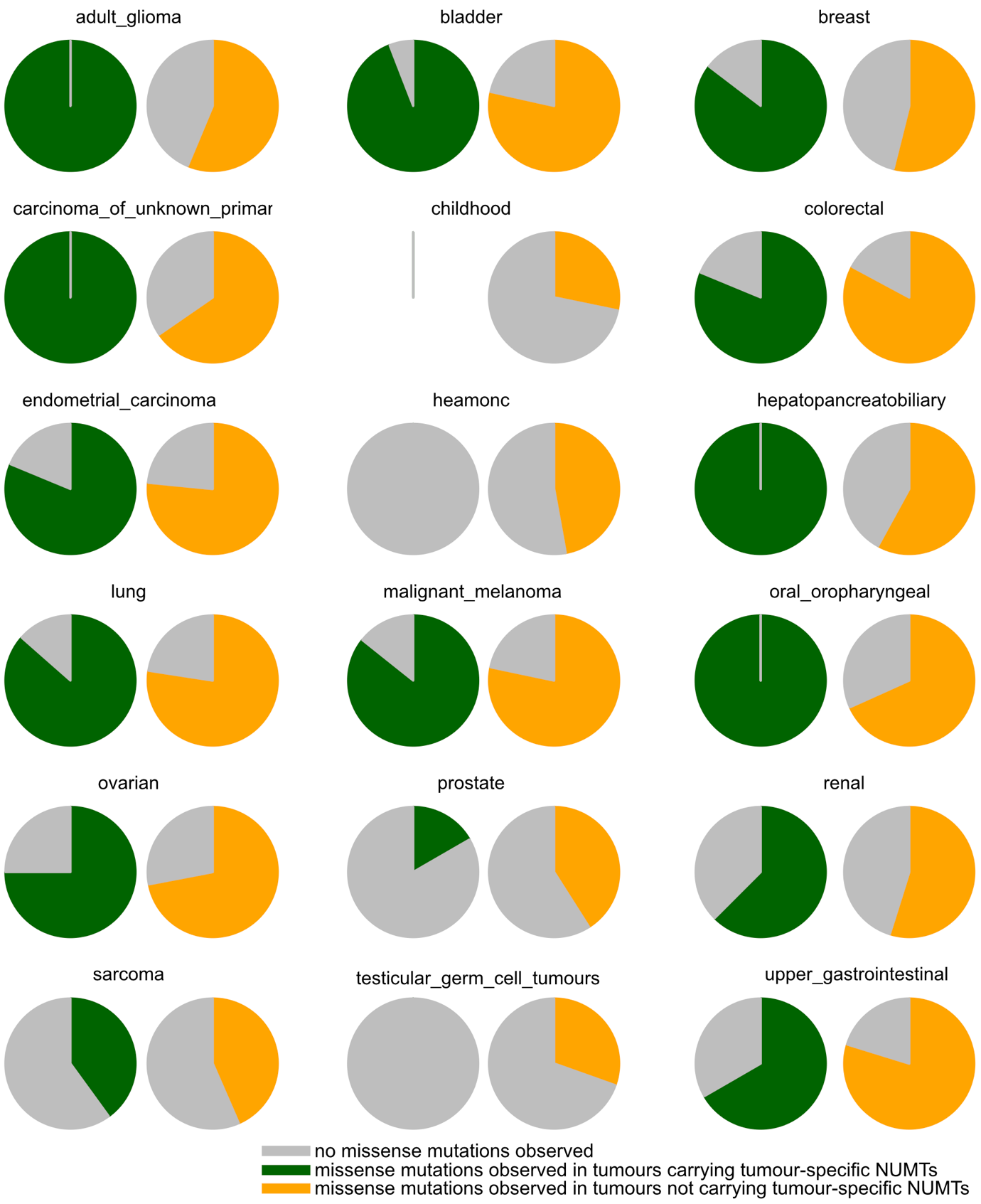
**

**Supplementary Figure 7. Pie charts of proportion of tumour samples carrying oncogene missense mutations in human DNA repair genes.** Tumour samples carrying tumour-specific NUMTs (left pie chart in each pair) and not carrying tumour-specific NUMTs (right pie chart in each pair) shown, separately.


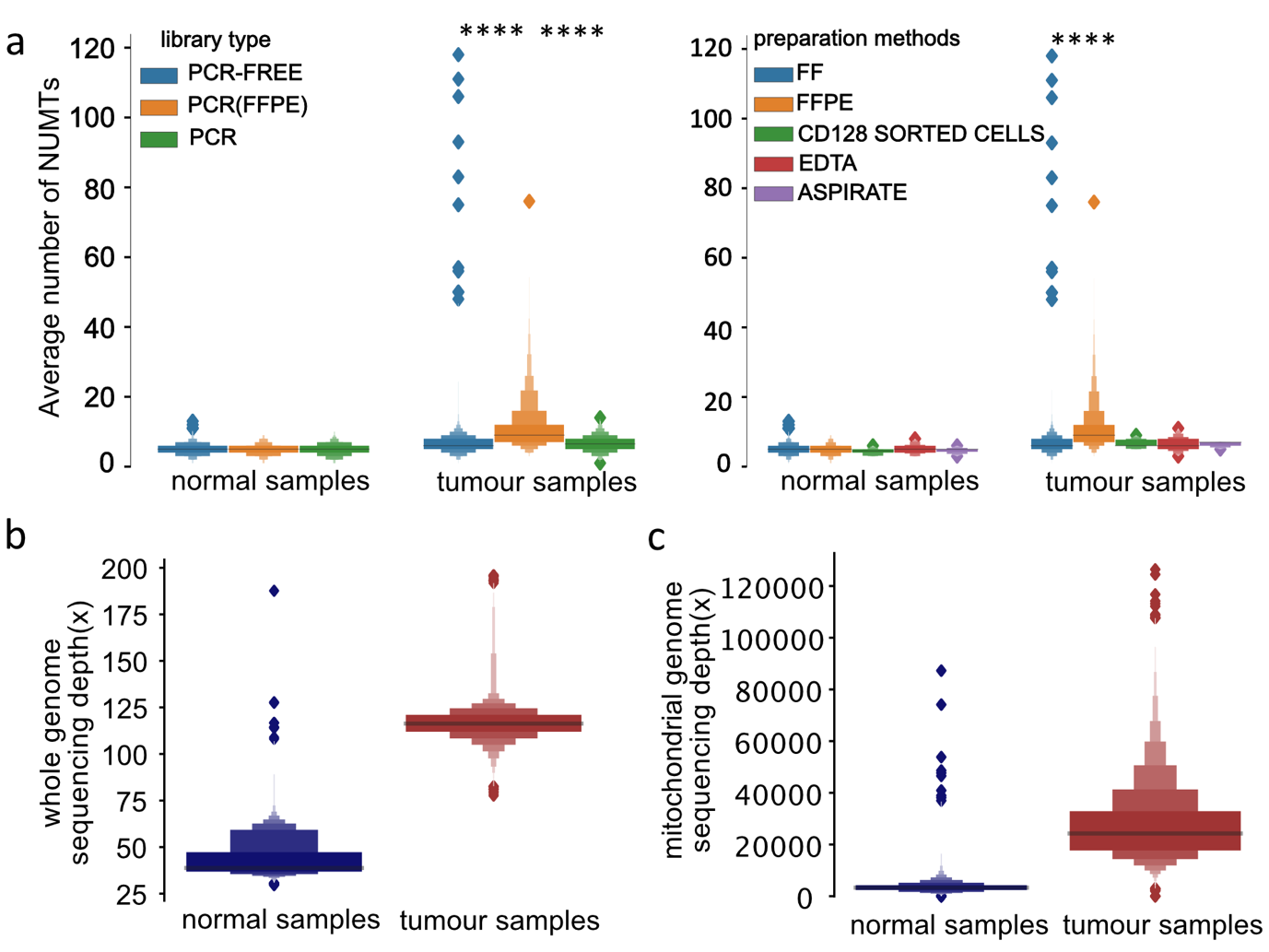


**Supplementary Figure 8.** NUMTs detected in the cancer genomes and the sequencing depth from the cancer genomes. **a.** Letter-value plots of the average number of NUMTs detected from different library types (left) and sample prepare methods (right). **b.** Letter-value plots of whole genome sequencing depth from cancer genomes, normal and tumour samples shown separately. **c.** Letter-value plots of mitochondrial genome sequencing depth from cancer genomes, normal and tumour samples shown separately. The middle line represents the median (50th percentile). Each successive level outward contains half of the remaining data. The first two sections out from the centreline contain 50% of the data. The next two sections contain 25% of the data. This continues until at the outlier level. The outliers are plotted as diamonds. P values # < 0.1, * < 0.05, <0.01 **, <0.001 ***, <0.0001 ****

**
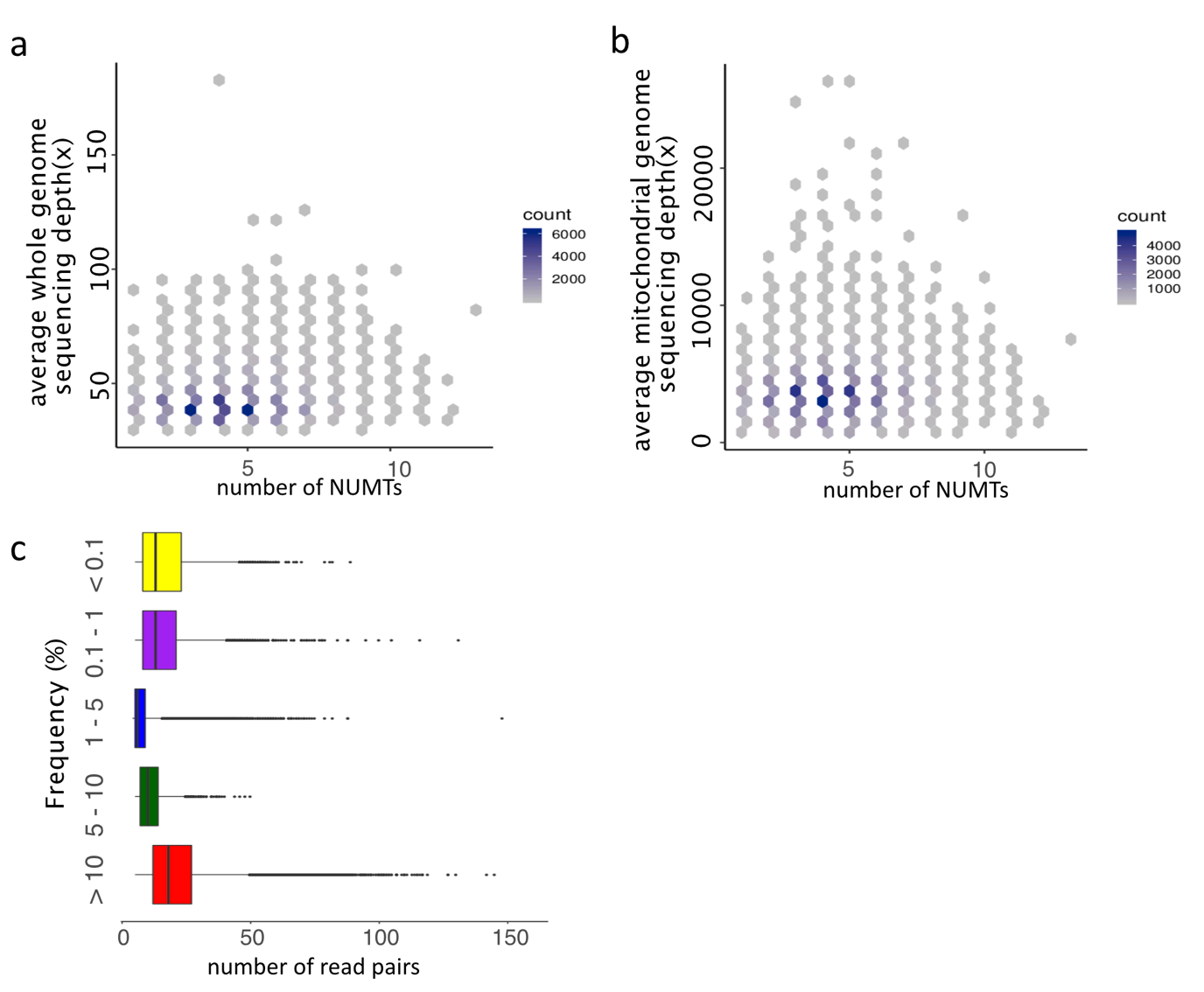
**

**Supplementary Figure 9. Correlation of the average number of NUMTs and the average sequencing depth. a.** Correlation of the average number of NUMTs and the average sequencing depth from whole genome sequence. **b.** Correlation of the average number of NUMTs and the average sequencing depth from mtDNA genome sequence. The colour corresponds to the number of individuals. **c.** Box plots show the number of discordant read pairs observed to detect NUMTs, grouped by the frequency of NUMTs. There was no significant difference between different frequency groups, suggesting NUMTs detection was not affected by the sequencing depth. >10% N = 220,756, 5-10% N = 5823, 1-5% N = 15,570, 0.1-1% N = 6794, <0.1% N = 5336. The middle “box” represents the median, lower and upper quartile of the data. The upper and lower whiskers represent the data outside the middle 50%. The dots represent the data outside 25% - 75% of the data.
