# Supplementary material for: Nuclear-embedded mitochondrial DNA sequences in 66,083 human genomes
Source: Nature. 2022 Oct 5;611(7934):105–14. doi: 10.1038/s41586-022-05288-7 (PMC9630118; doi:10.1038/s41586-022-05288-7)
Supplement: Supplementary file 2 — Reporting Summary [file 41586_2022_5288_MOESM2_ESM.pdf]

nature portfolio

Corresponding author(s): Patrick Chinnery 2022-01-00173

Last updated by author(s): Aug 6, 2022

Reporting Summary

Nature Portfolio wishes to improve the reproducibility of the work that we publish. This form provides structure for consistency and transparency in reporting. For further information on Nature Portfolio policies, see our Editorial Policies and the Editorial Policy Checklist.

Statistics

For all statistical analyses, confirm that the following items are present in the figure legend, table legend, main text, or Methods section.

n/a	Confirmed	
☐	☒	The exact sample size (*n*) for each experimental group/condition, given as a discrete number and unit of measurement
☐	☒	A statement on whether measurements were taken from distinct samples or whether the same sample was measured repeatedly
☐	☒	The statistical test(s) used AND whether they are one- or two-sided<br>*Only common tests should be described solely by name; describe more complex techniques in the Methods section.*
☐	☒	A description of all covariates tested
☐	☒	A description of any assumptions or corrections, such as tests of normality and adjustment for multiple comparisons
☐	☒	A full description of the statistical parameters including central tendency (e.g. means) or other basic estimates (e.g. regression coefficient) AND variation (e.g. standard deviation) or associated estimates of uncertainty (e.g. confidence intervals)
☐	☒	For null hypothesis testing, the test statistic (e.g. *F*, *t*, *r*) with confidence intervals, effect sizes, degrees of freedom and *P* value noted<br>*Give P values as exact values whenever suitable.*
☒	☐	For Bayesian analysis, information on the choice of priors and Markov chain Monte Carlo settings
☒	☐	For hierarchical and complex designs, identification of the appropriate level for tests and full reporting of outcomes
☐	☒	Estimates of effect sizes (e.g. Cohen's *d*, Pearson's *r*), indicating how they were calculated

Our web collection on statistics for biologists contains articles on many of the points above.

Software and code

Policy information about availability of computer code

Data collection	The sequence data included in this study is available in Genomics England Research Environment.
Data analysis	ISAAC (viSAAC-03.16.02.19); Strelka (v2.4.7); Manta (v0.28.0); Canvas (v1.3.1); PLINK(v1.9); BCFTools (v1.3.1); SAMtools (v1.9); MToolBox (v1.0); VarScan2; HaploGrep2; BLAT; bedtools (v2.19.1); R (v.3.6 to v.4.0); minimap2(v2.17); Nanoplot (v1.26.0); Nanopolish (v0.13.3); Python3; Circos (v0.69); IGV (v2.5); Shiny (v1.7.1).VerifyBamID (v1.1.3); ConPair (v0.2); R Package UMAP (v0.2.7.0); R Package M3C (v1.18); samblaster (v0.1.25); blat (v3.5); bedtools (v2.19.1); CAP3; BioPython (v1.77); Matplotlib(v3.3.1)<br>Custom code used in the study is available at: https://github.com/WeiWei060512/NUMTs-detection.git.<br>The software and methods used to do the analysis are all cited and described in the manuscript.

For manuscripts utilizing custom algorithms or software that are central to the research but not yet described in published literature, software must be made available to editors and reviewers. We strongly encourage code deposition in a community repository (e.g. GitHub). See the Nature Portfolio guidelines for submitting code & software for further information.

Data

Policy information about availability of data

All manuscripts must include a data availability statement. This statement should provide the following information, where applicable:

- Accession codes, unique identifiers, or web links for publicly available datasets
- A description of any restrictions on data availability
- For clinical datasets or third party data, please ensure that the statement adheres to our policy

The sequence data is available for analysis through the Genomics England data warehouse https://www.genomicsengland.co.uk/understanding-genomics/data/; Homo Sapiens NCBI GRCh38 assembly can be found at https://www.ncbi.nlm.nih.gov/assembly/;

GENCODE v29 can be found at https://www.gencodegenes.org/human/release_29.html;
Human genome annotation files can be found at https://hgdownload.soe.ucsc.edu/goldenPath/hg38/database/;
The ancestral mitochondrial sequences from Chimpanzee can be found at https://www.ensembl.org/Pan_troglodytes/Info/Index;

Field-specific reporting

Please select the one below that is the best fit for your research. If you are not sure, read the appropriate sections before making your selection.

☒ Life sciences ☐ Behavioural & social sciences ☐ Ecological, evolutionary & environmental sciences

For a reference copy of the document with all sections, see nature.com/documents/nr-reporting-summary-flat.pdf

Life sciences study design

All studies must disclose on these points even when the disclosure is negative.

Sample size	We studied 68,348 genomes in Genomics England Rare Disease Project and 26,488 cancer genomes from Genomics England Cancer Project. After all quality control steps, we included 53,574 genomes in Rare Disease Project and 12,509 tumour-normal tissue pairs in Cancer Project. We analysed all of the available data at the time we started this study, and reported the results of our statistical analyses with confidence intervals.
Data exclusions	We excluded the genomes aligned to the Homo Sapiens NCBI hg19 assembly, and failed either whole genome sequencing QCs or mitochondrial genome QCs. The details are described in the manuscript - Methods.
Replication	Replication was not possible. We used all available data in our primary analysis.
Randomization	We performed an observational study on all available data. Randomisation was not appropriate because our study design did not involve experimental interventions.
Blinding	We performed an observational study. No experimental interventions were performed, so blinding was not necessary

Reporting for specific materials, systems and methods

We require information from authors about some types of materials, experimental systems and methods used in many studies. Here, indicate whether each material, system or method listed is relevant to your study. If you are not sure if a list item applies to your research, read the appropriate section before selecting a response.

Materials & experimental systems

n/a	Involved in the study	
☒	☐	Antibodies
☒	☐	Eukaryotic cell lines
☒	☐	Palaeontology and archaeology
☒	☐	Animals and other organisms
☐	☒	Human research participants
☒	☐	Clinical data
☒	☐	Dual use research of concern

Methods

n/a	Involved in the study	
☒	☐	ChIP-seq
☒	☐	Flow cytometry
☒	☐	MRI-based neuroimaging

Human research participants

Policy information about studies involving human research participants

Population characteristics	We studied 68,348 genomes in Genomics England Rare Disease Project and 26,488 cancer genomes from Genomics England Cancer Project. After all quality control steps, we included 25,436 male and 28,138 females aged from 0 to 99y in Rare Disease Project (Extended Data Fig.1a&b) and 12,509 tumour-normal tissue pairs from 21 different cancer types in Cancer Project (Extended Data Fig.6a&b). More information can be found in the website https://www.genomicsengland.co.uk/about-genomics-england/the-100000-genomes-project/.
Recruitment	The Genomics England 100,000 Genomes Rare Disease Project enrolled people with a high likelihood or clear evidence of a rare inherited disorder. More information can be found in the website https://www.genomicsengland.co.uk/about-genomics-england/the-100000-genomes-project/information-for-gmc-staff/rare-disease-documents/rare-disease-eligibility-criteria/. Genomics England Cancer Project recruited patients with conditions corresponding to the eligibility criteria. The details can be found in the website https://www.genomicsengland.co.uk/about-genomics-england/the-100000-genomes-project/information-for-gmc-staff/cancer-programme/eligibility/
Ethics oversight	Ethical approval was provided by the East of England Cambridge South national research ethics committee under reference

Ethics oversight	number: 13/EE/0325, with participants providing written informed consent for this approved study. All consenting participants in the Rare Disease arm of the 100,000 Genomes Project were enrolled via thirteen centres in the National Health Service covering all NHS patients in England.

Note that full information on the approval of the study protocol must also be provided in the manuscript.
